# Neoplastic pathology at the crossroads between neck imaging and cardiothoracic imaging

**DOI:** 10.1186/s13244-020-00879-2

**Published:** 2020-07-08

**Authors:** Patricia E. Melendez, Trinh T. Nguyen, Alok A. Bhatt, Katherine Kaproth-Joslin

**Affiliations:** 1 University of Rochester Medical Center, 601 Elmwood Ave, Box 648, Rochester, NY 14642 USA; 2 Mayo Clinic, 4500 San Pablo Road, Jacksonville, FL 32224 USA

**Keywords:** Thoracic inlet, Head and neck imaging, Thoracic imaging, Radiology

## Abstract

The thoracic inlet is located at the crossroads between imaging of the neck and the chest. Its location is an important anatomic landmark, serving as the central conducting pathway for many vital structures extending from the neck into the chest and vice versa. Many critical body systems, including the respiratory, lymphatic, neurologic, enteric, musculoskeletal, endocrine, and vascular systems, are located within this region. Neoplasms, both benign and malignant, can arise in any of the body systems located in this area. Due to the small size of this anatomic location, pathology is easily overlooked and imagers should be aware of the imaging appearance of these neoplasms, as well as which imaging modality is the most appropriate for neoplasm evaluation. This article will present an image rich, system-based discussion of the neoplastic pathology that can occur in this region. The anatomy of the thoracic inlet and the non-neoplastic pathology of the thoracic inlet have been covered in our companion article.

## Key points

- The thoracic inlet is an important anatomic region from which various neoplastic pathologies can arise.
- It contains many vital body systems such as the respiratory, lymphatic, neurologic, enteric, musculoskeletal, endocrine, and vascular systems, allowing for the development of a system based approach to the review of the thoracic inlet.
- Many of these findings can be subtle and easily overlooked as the thoracic inlet is located at the crossroads between different imaging specialties.

## Introduction

The thoracic inlet is located at the crossroads between the cross-sectional imaging of the neck and chest. Its location is an important anatomic landmark, serving as the central conducting pathway for many vital structures extending from the neck into the chest and vice versa. This region of the body is typically located on the first or last set of images obtained for chest or neck imaging respectively. Because of this, there is a high chance for this region being over looked during imaging interpretation, especially if both the chest and the neck are being read independently by different subspecialty radiologists, as the neck imager may feel that it is part of the chest territory and the chest imager may feel that it is part of the neck territory. In addition, confident interpretation of this region can be difficult as many radiologists are fellowship trained in either neuroradiology or cardiothoracic/body imaging, but not both specialties.

When evaluating for neoplastic lesions of the thoracic inlet, it is important to tackle this region with a methodical plan in order to reduce the chance of missing clinically important findings. We propose a system-based approach to the imaging of the thoracic inlet focusing on the body systems present in this region. The systems to consider in this location are: respiratory, lymphatic, neurologic, enteric, musculoskeletal, endocrine, and vascular. Both benign and malignant neoplasms can arise from each of these systems (Table 1). Imagers must be aware how lesions from different systems in the thoracic inlet may present on different modalities and which modality would be the most optimal for initial evaluation, follow-up, and treatment response. This article presents an image rich systematic discussion of the neoplastic pathology that can occur in this region focusing on a system-based approach using different imaging modalities, including plain film, computed tomography (CT), magnetic resonance (MR), and ultrasound (US) imaging. The anatomy of the thoracic inlet, review of imaging modalities, and the non-neoplastic pathology of the thoracic inlet have been covered in our companion article [1]. 
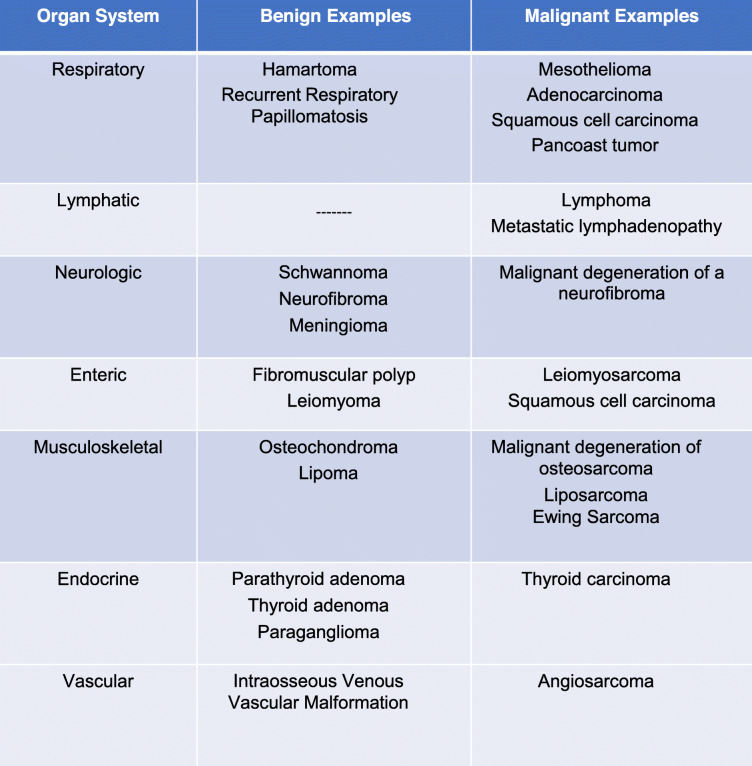

**Table 1:** Representative neoplastic pathology of the thoracic inlet

Organ System	Benign Examples	Malignant Examples
Respiratory	Hamartoma Recurrent Respiratory Papillomatosis	Mesothelioma Adenocarcinoma Squamous cell carcinoma Pancoast tumor
Lymphatic	-------	Lymphoma Metastatic lymphadenopathy
Neurologic	Schwannoma Neurofibroma Meningioma	Malignant degeneration of a neurofibroma
Enteric	Fibromuscular polyp Leiomyoma	Leiomyosarcoma Squamous cell carcinoma
Musculoskeletal	Osteochondroma Lipoma	Malignant degeneration of osteosarcoma Liposarcoma Ewing Sarcoma
Endocrine	Parathyroid adenoma Thyroid adenoma Paraganglioma	Thyroid carcinoma
Vascular	Intraosseous Venous Vascular Malformation	Angiosarcoma

## Respiratory system

Respiratory system lesions can arise from the airways, pulmonary parenchyma, or pleural surface. Careful evaluation of the lung apices and airways located at the level of the thoracic inlet can reveal many important neoplastic etiologies, both benign and malignant. Some of the more commonly encountered thoracic inlet neoplasms of the respiratory system include hamartoma, recurrent respiratory papillomatosis, mesothelioma, adenocarcinoma, squamous cell carcinoma, and Pancoast tumor.

### Hamartoma

**Fig. 1:**
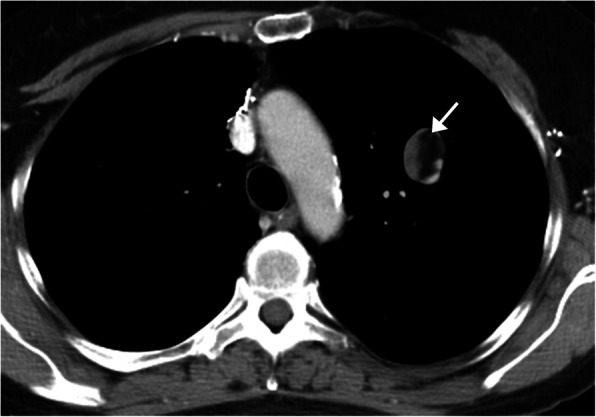
Hamartoma. CT chest with contrast demonstrating the pathognomonic finding of intrinsic fat within a pulmonary hamartoma of the left upper lobe (arrow)

Pulmonary hamartomas are one of the most common benign pulmonary tumors, accounting for up to 3% of all lung neoplasms and 6% of all solitary pulmonary nodules [2, 3]. The majority are asymptomatic and discovered incidentally on routine imaging. Hamartomas are slow growing and are heterogeneous both histologically and radiologically, containing cartilage, fat, muscle, fibroblastic tissue, and/or myxomatous tissue [4]. The majority are located within the periphery of the lung and are < 4 cm in diameter, but can measure up to 10 cm. CT is the imaging modality of choice to identify the fat and calcifications classically present. The visualization of intrinsic fat, measuring between − 40 and − 120 Hounsfield units (HU) in a slow growing/non-growing nodule is pathognomonic of a hamartoma (Fig. 1). The lesion typically is well-circumscribed with smooth or gently lobulated borders and can heterogeneously enhance [3]. Some hamartomas will demonstrate classic clumps of popcorn-like calcification [5]. Occasionally, larger lesions will demonstrate low grade uptake on 2-[fluorine-18] fluoro-2-deoxy-d-glucose (FDG)-positron emission tomography (FDG-PET)/CT imaging [3].

### Recurrent respiratory papillomatosis

**Fig. 2:**
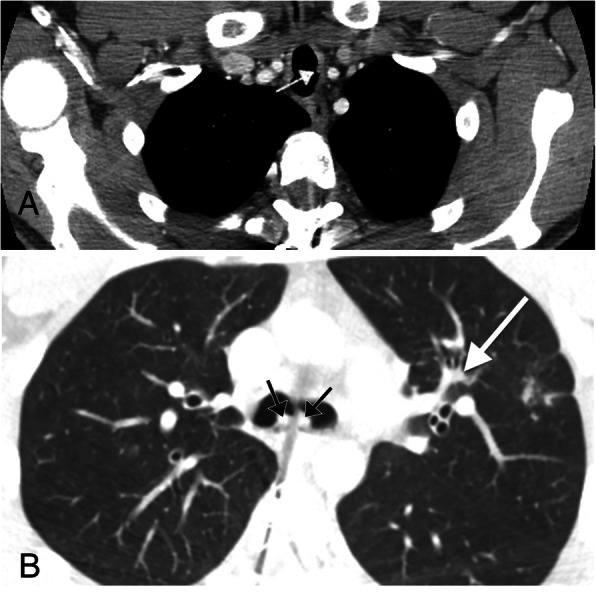
Recurrent respiratory papillomatosis. **a** Axial chest CT showing a soft tissue attenuation polyp in the lateral wall of the upper thoracic trachea (arrow). **b** Axial CT image of the same patient at the level of the carina showing two additional soft tissue attenuation polyps (black arrows). This image also demonstrates bronchiectasis in the left upper lobe which occurred secondary to luminal obstruction of a distal airway secondary to a polyp (white arrow)

Recurrent respiratory papillomatosis is a chronic disease occurring in both children and adults of viral etiology secondary to mucosal human papilloma virus (HPV) (types 6 and 11) infection. The condition is characterized by proliferation of multiple squamous papillomas arising from the respiratory epithelium, with the lesions more commonly found in the larynx as compared to the tracheobronchial tree [6]. CT is the imaging modality of choice to evaluate these lesions, with the lesions presenting as a solid or cavitary wart-like lesion arising from the surface of the airway [6] (Fig. 2). Luminal narrowing of the airway can lead to atelectasis, bronchiectasis, and mucous plugging [7] (Fig. 2b). Small thin-walled cysts with adjacent nodules may be present in the pulmonary parenchyma, typically measuring between 2–3 mm in size. Malignant degeneration into squamous cell carcinoma should be considered if a lesion demonstrates rapid growth, with particular attention to the nodular component of a predominantly cystic lesion.

### Mesothelioma

**Fig. 3:**
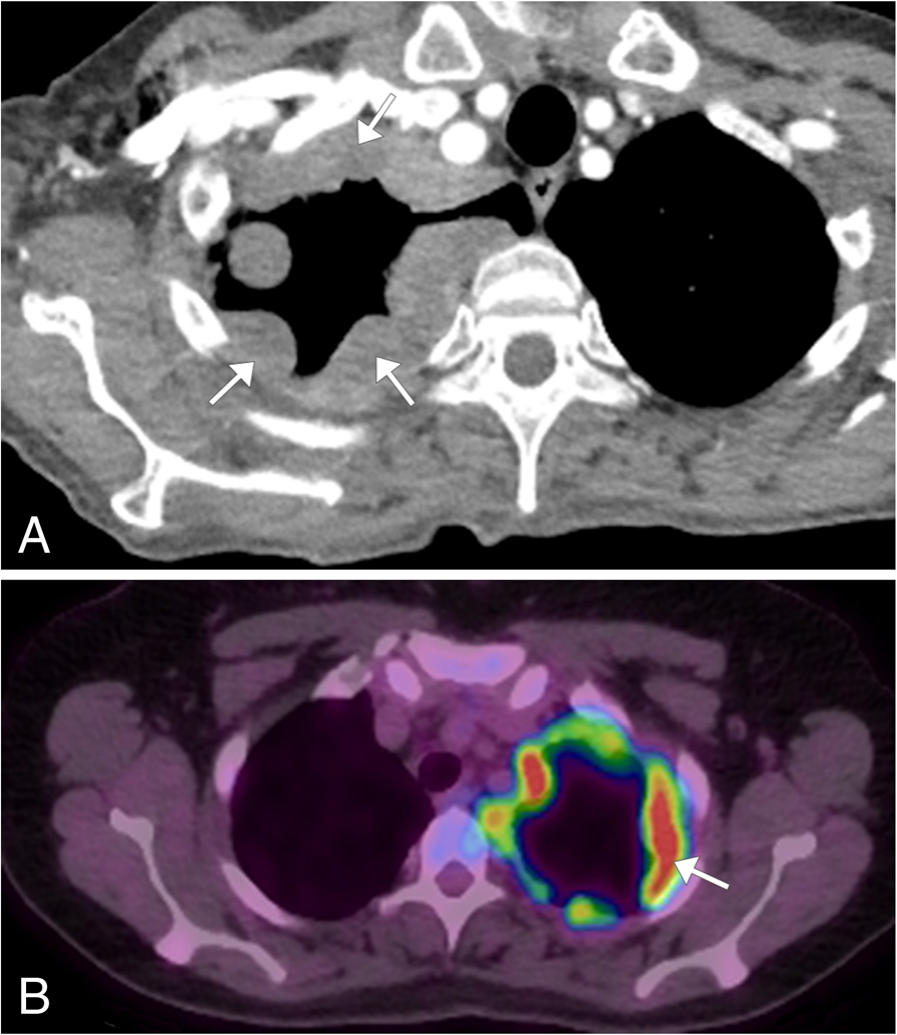
Mesothelioma. **a** Axial chest CT showing marked nodular thickening of the pleura throughout the right pleural surface (arrows). **b** Axial FDG-PET/CT image of a companion case showing the hypermetabolic activity of the nodular pleural lesions (arrow) in the setting of biopsy proven mesothelioma

Mesothelioma is a malignant tumor of the mesothelial cells and is the most common primary pleural malignancy and second most common overall pleural malignancy after metastasis. It is strongly associated to prior asbestos exposure and has a higher incidence in men (ages 50–70 years old). Mesothelioma has a poor prognosis due to its aggressive nature. Patients commonly present with non-pleuritic chest pain and dyspnea. On radiographs, a unilateral pleural effusion is commonly seen and may be the only finding. Pleural-based thickening or masses can be seen in 45–60% of cases [8]. Chest CT is the imaging modality of choice to evaluate mesothelioma, showing the size of the tumor, associated lymphadenopathy, and local invasion or extra-thoracic spread if present [9]. The pleural thickening can be nodular or smooth in configuration, and mesothelioma should be suspected if the pleural thickening is greater than 1 cm (Fig. 3a). Calcifications related to asbestos disease can be seen in 20% of mesothelioma cases and if located in the tumor, it typically represents engulfment of a previously present pleural plaque and not necessarily intrinsic calcification of the tumor [8]. MRI is not routinely used for evaluation but can provide more precise staging information in certain circumstances, with a higher sensitivity than CT in detecting invasion of the chest wall, mediastinum, and diaphragm [9]. FDG-PET/CT is used as an adjunctive study both for staging and diagnosis, and the tumor is avidly hypermetabolic (Fig. 3b). This modality can be used to plan image-guided and surgical biopsies, as well as evaluating treatment response. In patients who have undergone talc pleurodesis as treatment of malignant pleural mesothelioma, the treated areas will be FDG avid which makes the differentiation between post-treatment changes and disease progression difficult on PET imaging [10]. Because of this, MRI is often the preferred modality to follow for disease progression in these patients.

### Lung cancer

#### Adenocarcinoma

**Fig. 4:**
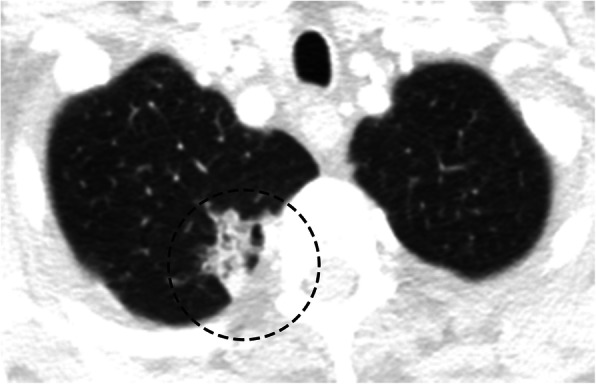
Adenocarcinoma of the lung. Axial image of a CT chest demonstrating an irregular shaped ground glass semisolid lesion in the apex of the right lung (dashed circle) in the setting of biopsy proven invasive adenocarcinoma

Adenocarcinoma is currently the most common histological subtype of lung cancer in most countries, making up half of all lung cancers [11, 12] If symptomatic, patients commonly present with cough, hemoptysis, dyspnea, chest pain, and non-resolving air-space opacity on imaging [13]. Adenocarcinoma of the lung exists on a spectrum, with its subtypes subdivided based on extent of invasion, a finding that is reflected in its imaging characteristics [12]. Adenocarcinoma in situ (AIS) is a noninvasive lesion measuring less than 3 cm that grows along the walls of the alveoli (lepidic growth) with no evidence of stromal, vascular, or pleural invasion [12]. Imaging of AIS will show a groundglass lesion without a solid component. Minimally invasive adenocarcinoma (MIA) is a semisolid lesion measuring < 3 cm with a lepidic dominant groundglass pattern on imaging and a small solid invasive component measuring up to 5 mm in its largest dimension [12, 14]. Invasive adenocarcinomas can range from a predominant lepidic pattern to a complex solid invasive growth pattern [12], with imaging ranging from a pure groundglass lesion larger than 3 cm, to a semisolid lesion with a solid component greater than 5 mm, to a fully solid lesion [14] (Fig. 4). CT is the ideal modality to evaluate these lesions and assess extent of disease for proper staging. In addition, it is common for synchronous and nonsynchronous primary adenocarcinomas to be present or develop over time. PET-CT may be useful in diagnosis and staging, as well as evaluating for treatment response. AIS and MIA typically demonstrates uptake < 1.95 SUV, while the more aggressive and invasive adenocarcinomas typically have an uptake of > 1.95 SUV [15].

#### Squamous cell carcinoma

**Fig. 5:**
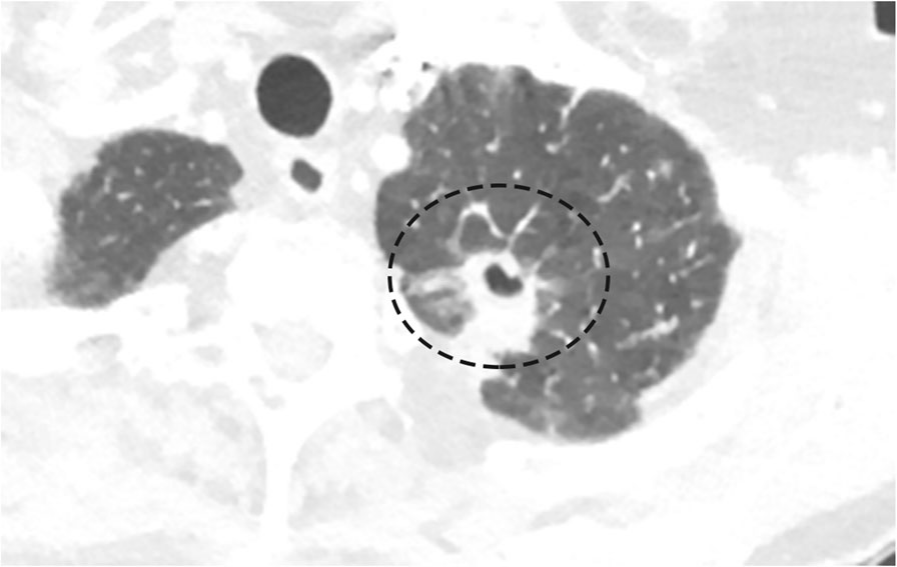
Squamous cell carcinoma of the lung. Axial chest CT image which demonstrates a spiculated lesion with a central cavitation in the left lung apex (dashed circle) in the setting of biopsy proven squamous cell carcinoma

Squamous cell carcinoma (SCC) is the second most common form of lung cancer, comprising 30% of all lung cancers and has a strong association with smoking [16]. This cancer is characterized by the presence of keratinization and/or intercellular bridging by tumor cells [16]. The majority of these lesions arise from the tracheobronchial tree (approximately two thirds), and the remaining will present as a solitary nodule [17]. Approximately, 80% of patients present with clinical symptoms similar to adenocarcinoma, with cough, chest pain, hemoptysis, dyspnea, and non-resolving opacity on imaging [13]. Large lesions can have extensive necrosis and cavitation [12]. CT with contrast is useful for the evaluation of tumor size, sometimes difficult to measure if there is significant post obstructive atelectasis from tumor also presesnt, and tumor characteristics, such as necrosis with or without cavitation, spiculation, and wall thickness in cavitary lesions (Fig. 5). CT also evaluates for lymphadenopathy, invasion into the adjacent structures, and identifying distant metastatic disease [18]. FDG-PET/CT is useful for staging and for assessing tumor response to treatment, with an SUV of > 2.5 indicating an increased likelihood of malignancy and an SUV > 5 being concerning for an invasive tumor [19]. It is important to remember that other inflammatory etiologies, such as tuberculosis, can be both FDG avid and can also present as an irregular lung lesions, including cavitary lesions with thick margins, and can be confused with malignancy and need to be excluded as false positives [20]. MR can be used as a complimentary study to evaluate for local invasion into adjacent structures, including the thoracic inlet, especially the brachial plexus and chest wall.

#### Pancoast tumor

**Fig. 6:**
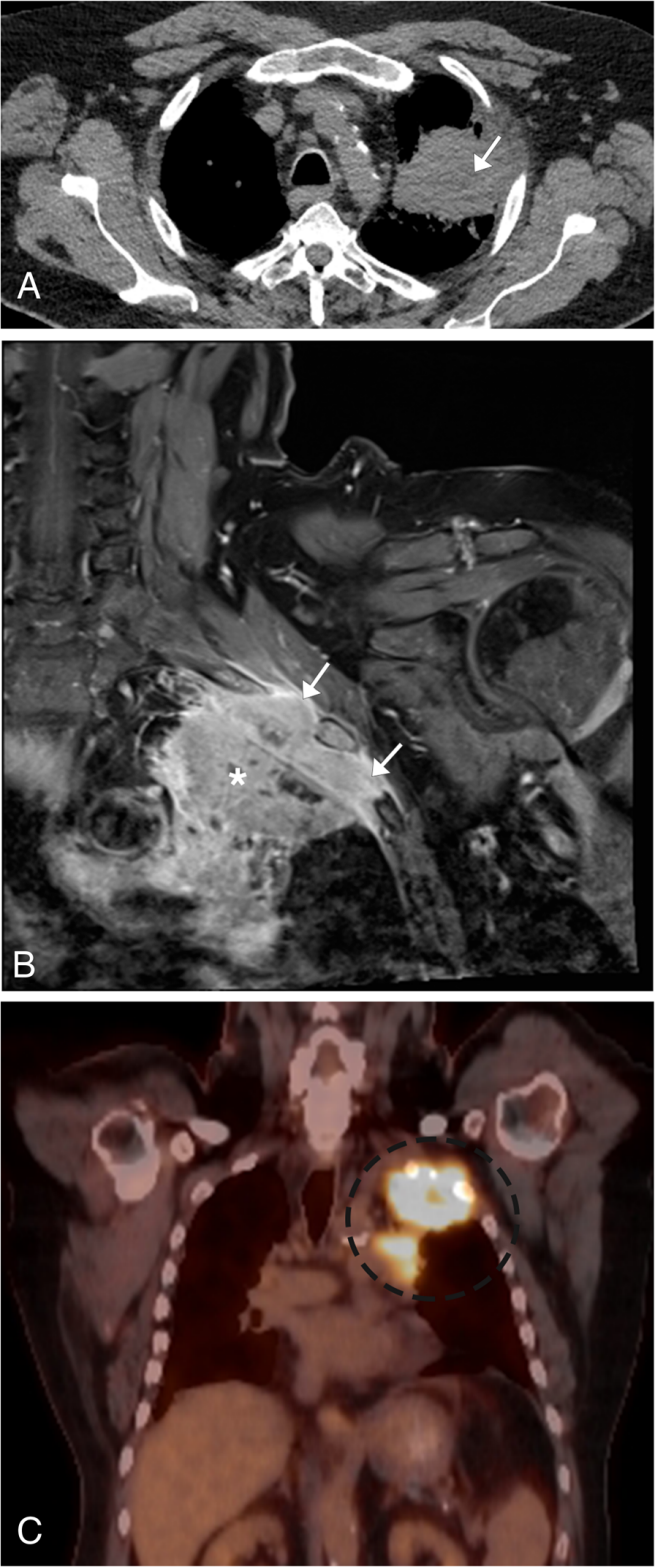
Pancoast Tumor. **a** Axial non-contrast CT of the chest demonstrates a large solid irregular mass in the left lung apex (arrow). **b** Coronal T1WI MR post contrast shows the enhancing mass (*) invading into the chest wall with involvement of the adjacent first to third left ribs (arrows). **c** Coronal FDG-PET/CT image demonstrates that the left lung apex mass is avidly hypermetabolic (dashed circle)

Pancoast tumor, also known as a superior sulcus tumor, is a non-small cell carcinoma that originates in the lung apex and invades the adjacent chest wall tissues. Historically, squamous cell carcinomas were once the most common histological form of this tumor; however, adenocarcinomas are now the predominant tumor type. Classically, this tumor presents with Pancoast syndrome (occurring in only 25% of patients), characterized by Horner’s syndrome (ipsilateral facial anhidrosis, pupillary miosis, and ptosis), shoulder pain, and C8-T2 radicular pain. The cause for this clinical presentation is invasion of the tumor into the brachial plexus and the sympathetic chain [21]. These lesions are hard to detect radiographically due to the apical location and often appear as a subtle soft tissue mass in the apex with or without destruction of the adjacent bone. CT is useful to confirm the presence of a mass in the lung apex, assess for associated osseous/chest wall involvement, and allows for accurate staging (Fig. 6a). MR is superior to CT in depicting tumor invasion of the thoracic wall, involvement of the brachial plexus, and extension of the tumor into the adjacent neuroforamina and spinal cord, and is considered a standard reference exam for evaluation of these tumors [22, 23] (Fig. 6b). The ideal planes to evaluate the superior extension on CT and MR imaging are the coronal and sagittal views [21]. As mentioned previously, these lesions will be hypermetabolic on PET-CT, and this imaging modality is useful in diagnosis and staging, as well as evaluating for treatment response (Fig. 6c).

## Lymphatic system

Neoplasms of the lymphatic system can arise from the lymph nodes or the lymphatic channels. In addition, the lymph nodes of the thoracic inlet are a common site for metastatic disease, including the classic Virchow nodule of the left neck base. Some of the more commonly encountered thoracic inlet neoplasms of the lymphatic system include lymphoma and metastatic lymph nodes.

### Lymphoma

**Fig. 7:**
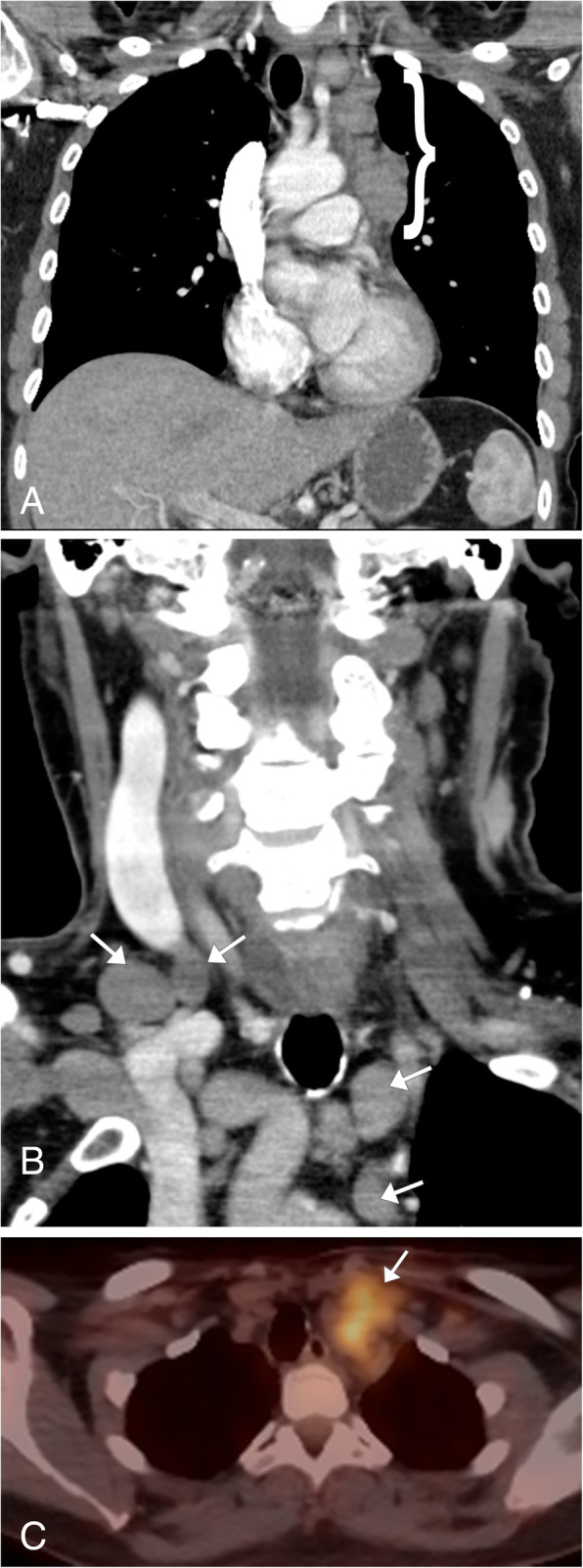
Lymphoma. **a** Coronal post contrast chest CT image with multiple enlarged left paratracheal and aortopulmonary window lymph nodes (bracket) in a patient with Hodgkin’s lymphoma. **b** Coronal CT post contrast image of the neck base shows multiple enlarged right neck base and left superior mediastinal lymph nodes in a patient with non-Hodgkin’s lymphoma (arrows). **c** Axial FDG-PET/CT image at the level of the thoracic inlet in the patient with Hodgkin’s lymphoma demonstrates hypermetabolic activity of the enlarged left paratracheal lymph nodes (arrow)

Lymphoma is a malignant lymphoproliferative disorder arising from lymphocytes or lymphoblasts. There are approximately 50 types of lymphoma which are divided based on histopathologic, immunohistochemical, cytogenetic, and molecular analysis [24]. Lymphoma is classically divided into Hodgkin’s (40%) and non-Hodgkin’s (60%) lymphoma. Hodgkin’s lymphoma, characterized by the histologic identification of Reed-Sternberg cells, follows a predictable contiguous spread along lymphatic pathways, with limited extra-nodal involvement. Non-Hodgkin’s lymphoma is a heterogeneous group of malignancies that have been divided into more than 20 subtypes based on cell of origin (B–T cell precursor), morphologic, and immunologic histology [25]. These tumors can have either contiguous or discontinuous nodal spread and are more likely to have extranodal involvement when compared to Hodgkin’s lymphoma. Patients will classically present with B-type symptoms, including night sweats, fever, and weight loss. Imaging will depend on the location and subtype of the lymphoma and if extranodal involvement is present. CT is useful in evaluating nodal and extranodal involvement, typically presenting with homogenous enlargement of the lymph nodes unless necrosis is present [24, 25] (Fig. 7a, b). FDG-PET/CT is the modality of choice for staging lymphoma, as this modality has greater sensitivity in evaluating the extent of disease and is a fundamental tool for the assessment in treatment response, and now incorporated within the Lugano criteria which assess lymphoma for treatment response [24] (Fig. 7c). Due to its superior soft tissue evaluation, MR can be useful to assess for central nervous system (CNS), vascular, and cardiac involvement. Lesions are commonly homogeneous on T1WI and homogeneous or heterogeneous signal on T2WI due to necrosis.

### Metastatic lymph nodes

**Fig. 8:**
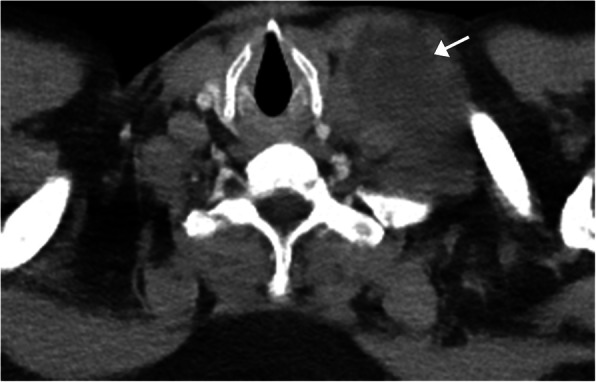
Metastatic lymph node. Axial contrast enhanced chest CT image shows a large ill-defined left supraclavicular lymph node with peripheral soft tissue attenuation and area of central hypoattenuation, likely representing central necrosis (arrow). This lymph node was consistent with metastatic disease in the setting of gastric carcinoma, a classic example of a Virchow’s node

Metastatic lymph nodes are commonly identified at the level of the thoracic inlet. The pattern of lymphatic flow can help predict the primary malignancy. For example, level II neck nodes typically correspond to a primary site in the pharynx, including tonsils, and level I neck nodes often are associated with the oral cavity [26]. There is a predilection of the primary tumors below the clavicle to metastasize in the deep cervical nodes in the left neck. This is because the thoracic duct (which drains the thorax, abdomen, and pelvis) joins the systemic venous system in the neck at the junction of the left subclavian and internal jugular veins [26]. Metastatic involvement in this node is known as Virchow’s node and should be suspected if an abnormal lymph node is identified in this location (Fig. 8). Suggestive features include enlargement of the lymph node measuring more than 1 cm in short axis, a rounded instead of oval contour, and loss of the normal fatty hilum. On physical exam, this finding is known as Troisier’s sign. Gastric carcinoma is the most common malignancy to spread to this left supraclavicular lymph node [27], however, can be seen with other primary cancers, including tumors of the CNS (glioblastoma multiforme, ependymoma, and oligodendroglioma), breast, lung, esophageal, and genitourinary tract (testicular, cervical, uterine, ovarian, bladder, and prostate). These lymph nodes can be evaluated with multiple modalities including US, MR, and CT, and abnormal lymph nodes may be found incidentally on imaging performed for other reasons. FDG-PET/CT may be utilized to evaluate these metastatic lymph nodes, and to look for the primary site of disease, both of which will show avid FDG uptake.

## Neurologic system

The signs and symptoms related to neurologic neoplasms of the thoracic inlet are dependent on which of the many important nerves that traverse this region is involved, including the brachial plexus, vagus nerve (recurrent laryngeal nerve branches), phrenic nerve, and sympathetic chain. Some examples of commonly encountered thoracic inlet neoplasms of the neurologic system include schwannoma, neurofibroma (without or with malignant degeneration), and meningioma.

### Schwannoma

**Fig. 9:**
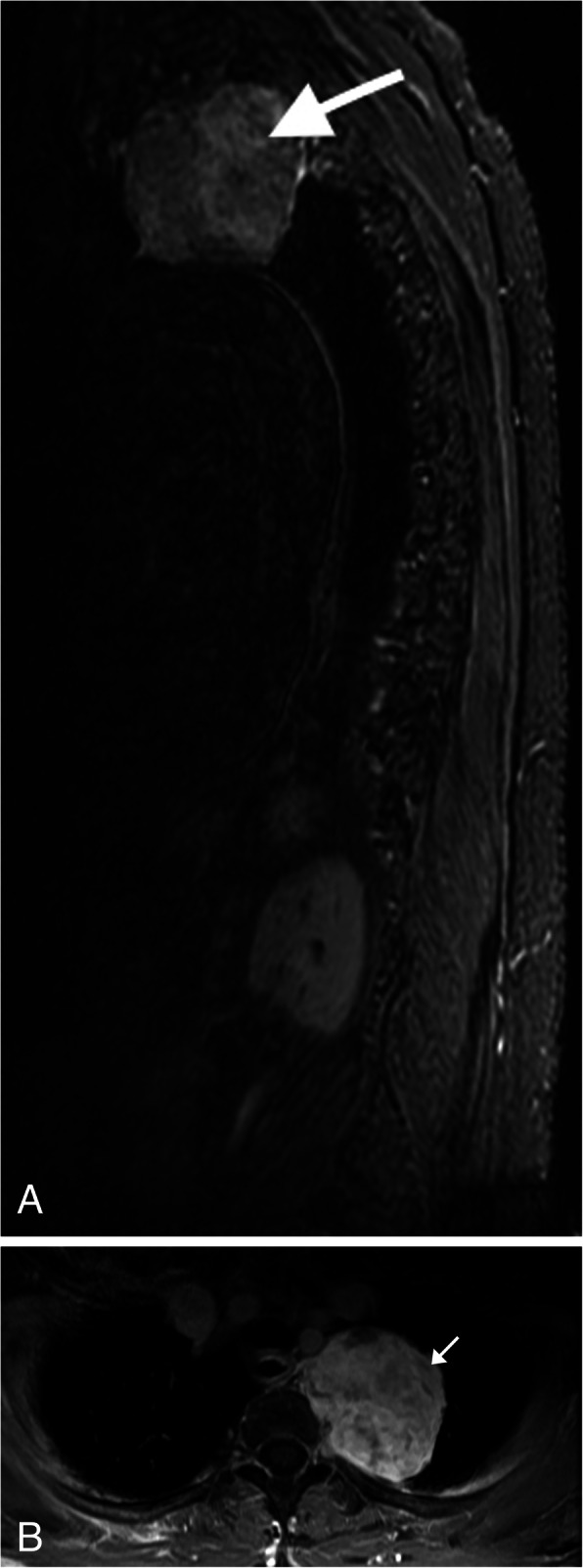
Schwannoma. **a** Sagital T2WI MR image demonstrates a well-circumscribed heterogeneous, predominantly hyperintense lesion arising from a right upper thoracic neural foramen. The lesion has multiple internal low-intensity ring-like structures (arrow), called the fascicular sign (representing fascicular bundles within the nerve). **b** Axial T1WI MR post contrast image of the thoracic spine in a companion case demonstrates heterogeneous enhancement of the lesion arising from the left T2 neuroforamina (arrow)

Schwannoma is a benign slow growing nerve sheath tumor composed of Schwann cell origin. These lesions can originate from any peripheral nerve in the body, with the majority of schwannomas in the head and neck arising from the vagus nerve [28]. The majority of these tumors are sporadic, but 10% will be associated with neurofibromatosis type 2 (NF2), in which multiple schwannomas may develop. CT imaging will show a well-demarcated mass, which is iso-attenuating to muscle prior to contrast administration, after which will demonstrate some degree of enhancement. MR imaging will demonstrate iso-hypointense signal relative to the spinal cord on T1WI and hyperintense signal on T2WI (Fig. 9a). The fascicular sign can be seen on T2WI characterized by low signal intensity in a ring-like pattern [29]. Different patterns of intense enhancement can be seen on post contrast images [28] (Fig. 9b).

### Neurofibromas

**Fig. 10:**
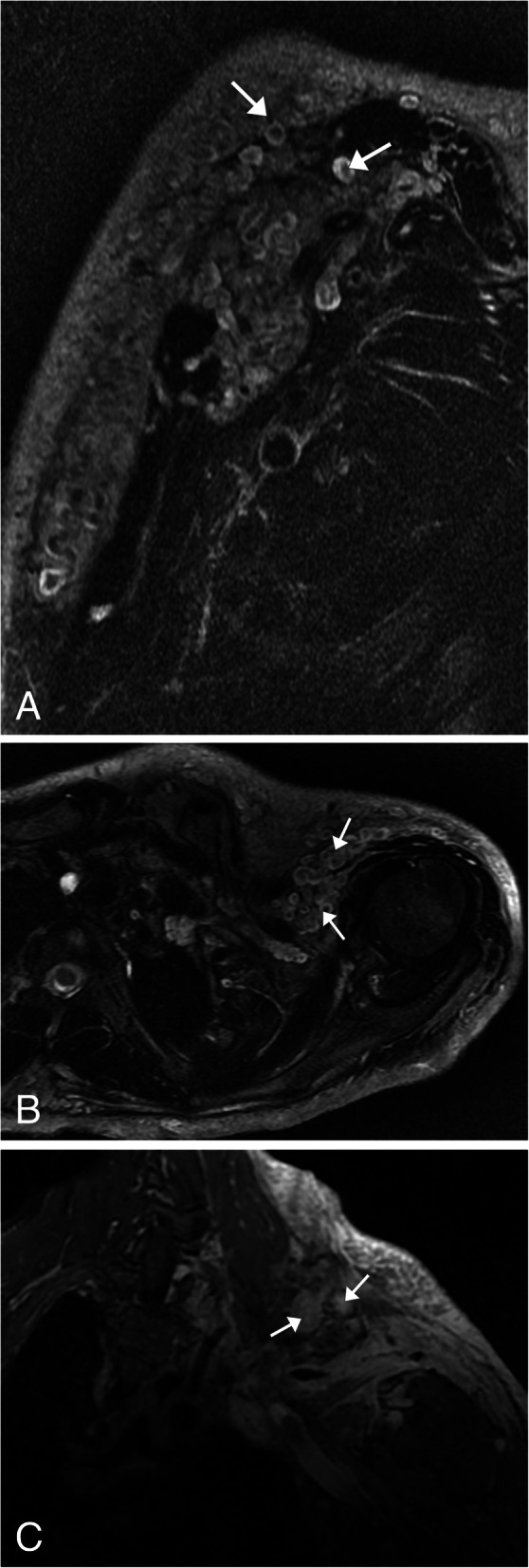
Neurofibroma. T2WI MR sagittal (**a**) and axial (**b**) images of the chest showing multiple round lesions with peripheral hyperintensity and central hypointensity, giving the classic appearance of the target sign (arrows). **c** Coronal T1WI MR post contrast demonstrates mild central enhancement with a non-enhancing periphery (arrows)

Neurofibroma is another benign peripheral nerve sheath tumor which has a strong association with neurofibromatosis type 1 (NF1). This tumor is composed of an unencapsulated proliferation of all nerve elements, and the tumor is intermixed and inseparable from the affected nerve [29]. This tumor can be subdivided into three types: localized, diffuse cutaneous, and plexiform, each with a unique imaging appearance. The modality of choice to evaluate these lesions is MRI, in which the lesion will appear hypointense on T1WI and intermediate to high intensity on T2WI. The target sign may be present, in which the lesion will demonstrate a low central signal from collagen deposition with a hyperintense rim when viewed in the short axis plane. Post contrast, the T2 hypointense region centrally will show some enhancement, and the periphery will remain unenhanced [29] (Fig. 10).

**Fig. 11:**
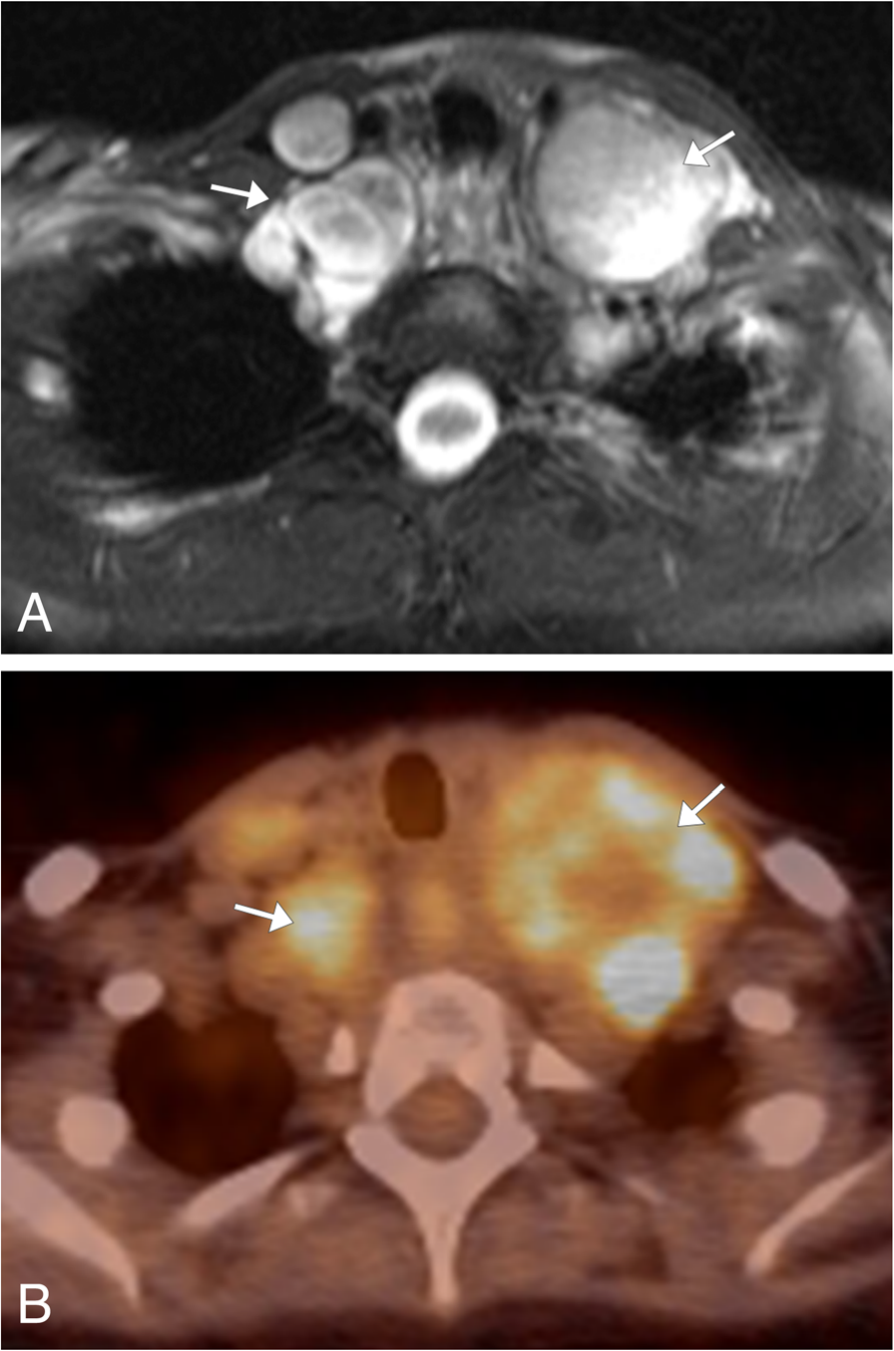
Malignant peripheral neural sheath tumor (MPNST). **a** Axial T2WI MR of the neck base demonstrating several round well-circumscribed large lesions which are predominantly hyperintense (arrows). The classic target sign on T2WI is lost. FDG-PET/CT axial (**b**) image shows these lesions to have heterogeneous increased metabolic activity (arrows)

Neurofibromas can undergo malignant degeneration and when this occurs, it is classified as a malignant peripheral neural sheath tumor (MPNST). These tumors typically occur in the deep soft tissues, and the most common sites are the sciatic nerve, brachial plexus, and sacral plexus [30]. These neoplasms account for 5–10% of the soft tissue sarcomas, most common in patients aged 20–50 years [31]. Patients may present with neurologic deficit secondary to impingement of the associated nerves [30]. On MRI, MPNST will appear as an invasive mass that is iso or hyperintense on T1WI and hyperintense on T2WI [31]. Heterogeneity can be present secondary to internal necrosis, hemorrhage, and cellularity [31]. The classic target sign of neurofibromas is absent [31] (Fig. 11a). On CT surveillance imaging, MPNST should be suspected if there is a change in size of a neurofibroma or heterogeneous attenuation is identified [31]. PET imaging will demonstrate heterogeneous hypermetabolism secondary to the presence of necrosis and hemorrhage [31] (Fig. 11 b).

### Meningioma

**Fig. 12:**
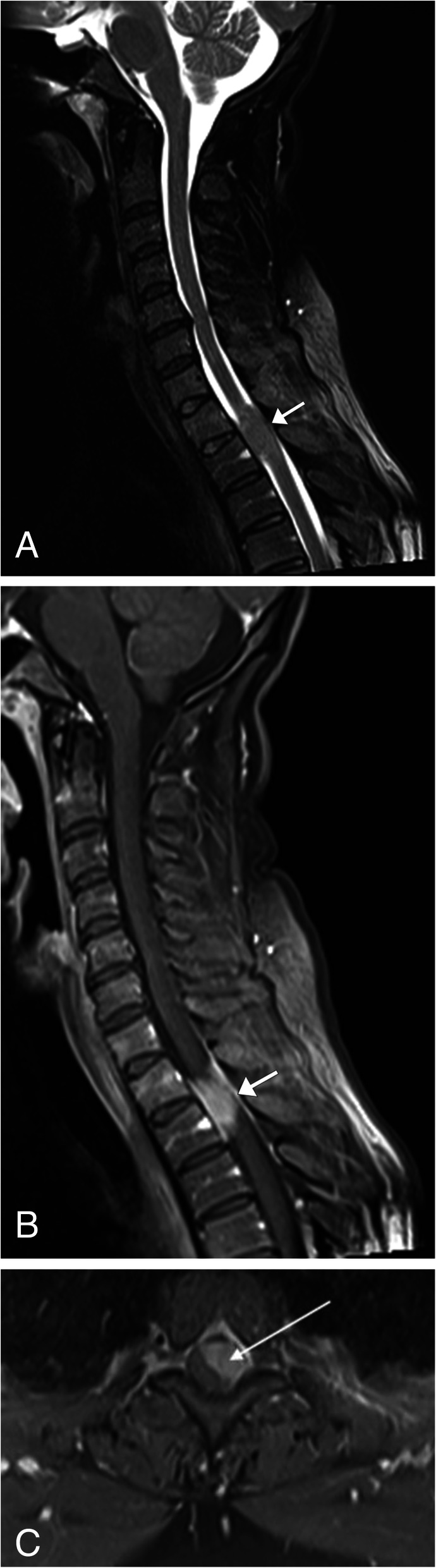
Meningioma. **a** Sagittal T2WI MR image of the cervical spine shows a well-circumscribed T2 hypointense lesion within the spinal cord at the level of T1–T2 (arrow). There is no evidence of increased T2 signal of the spinal cord to suggest edema. **b** Sagittal T1WI MR post contrast image and (**c**) axial TIWI post contrast of the cervical spine shows homogenous enhancement of the lesion. Note adjacent dural tail sign (short arrow). The axial image confirms the extramedullary location of the lesion (long arrow), as the cord is displaced to the right aspect of the spinal canal

Meningioma is one of the most common primary tumors of the spine, accounting for 25–46% of spinal neoplasms [32]. It is typically a benign tumor of non-glial cell origin that occurs more commonly in women [32, 33]. These lesions can be classified by location or subdivided by intradural/extradural involvement [33]. This is a slow growing tumor which may present with symptoms often related to mass effect, including radicular pain, sensory loss, and paresthesia [32]. The lesion is typically round or ovoid with a broad dural attachment, known as the dural tail. These lesions are difficult to see on non-contrast CT because they are similar in attenuation to the spinal cord; however, post contrast, the meningioma will demonstrate strong homogeneous enhancement making them easier to see. Calcifications can be seen in 1–5% of cases. The imaging modality of choice for these lesions is MRI, in which the meningiomas will appear isointense to the spinal cord on T1WI and have a varied appearance on T2WI ranging from isointense to hyperintense when compared to the spinal cord (Fig. 12). If the meningioma contains calcification, regions of low signal will be noted. Post contrast, imaging will demonstrate diffuse homogenous contrast enhancement, and the dural tail sign can be appreciated showing tapered thickening and enhancement of the dura adjacent to the tumor [32] (Fig. 12).

## Enteric

The cervical and thoracic portions of the esophagus are the most common source of enteric neoplasm in the thoracic inlet. Symptoms, when present, are often related to narrowing/obstruction of the esophagus, including dysphagia, cough, and chest pain. Some of the more commonly encountered thoracic inlet neoplasms of the enteric system include fibrovascular polyp, leiomyoma, and esophageal cancer.

### Fibrovascular polyp

**Fig. 13:**
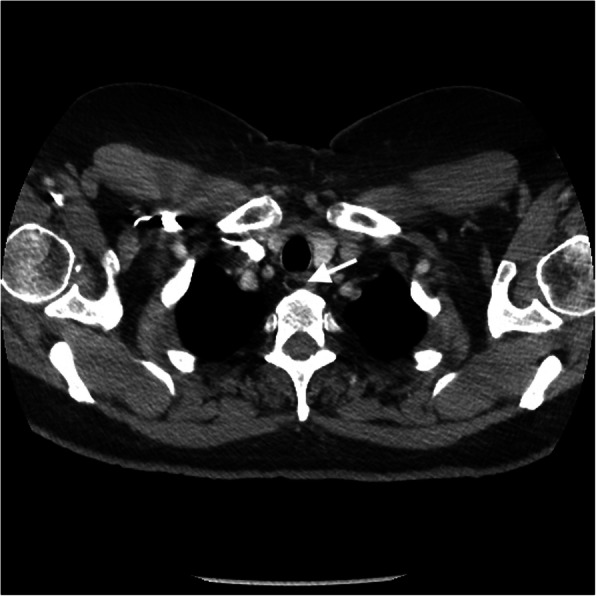
Fibrovascular polyp. Axial CT image of the superior thorax demonstrates a fat attenuation lesion in the right lateral esophageal wall in the upper esophagus (arrow) consistent with predominant adipose tissue

Fibrovascular polyp of the esophagus is a rare and benign mesenchymal tumor. The lesions are composed of fibrous, vascular, and adipose tissue covered by normal squamous epithelium. This lesion can be subdivided histologically by the types of tissue and it is composed of hamartoma, lipoma, and fibrolipoma [34]. The majority grow in the upper third of the esophagus, most commonly arising at the level of the cricopharyngeus muscle in the cervical esophagus. These intraluminal lesions grow gradually, often becoming large, and can elongate over a period of years and be dragged inferiorly by esophageal peristalsis. Patients may be asymptomatic or present with dysphagia or respiratory symptoms. The imaging study of choice is a fluoroscopic barium esophagram, which typically reveals a smooth, expansile, sausage-shaped mass in the upper to middle third of the esophagus. CT imaging will be dependent on the composition of the polyp, ranging from fat density if it contains a large amount of adipose tissue to heterogeneous attenuation in the setting of abundant fibrovascular tissue [35] (Fig. 13). It is important to note, however, that CT imaging can be limited if the esophageal lumen is not distended, and these lesions can be easily overlooked as they are similar in appearance to the adjacent mucosa [34].

### Leiomyoma

Leiomyoma is the most common submucosal tumor of the esophagus. These lesions contain intersecting bands of smooth muscle and fibrous tissue surrounded by a well-defined capsule. They are usually located at the level of the thoracic esophagus below the level of the aortic arch. While typically asymptomatic, if the lesion is large, patients can present with dysphagia, obstruction, cough, and chest pain. As with esophageal polyp evaluation, the study of choice for evaluation is a barium esophagram. Imaging will demonstrate a smooth rounded submucosal mass, and the sharp division between the tumor and the esophageal lumen creates an abrupt angle on lateral images [36].

**Fig. 14:**
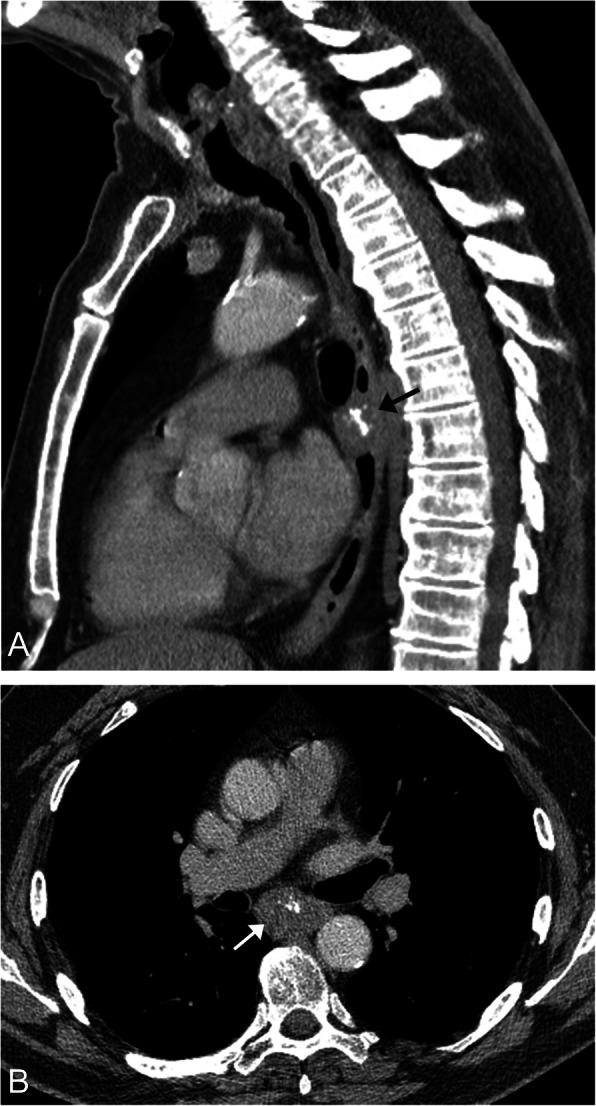
Leiomyoma. Sagittal (**a**) and axial (**b**) CT image of the chest demonstrates an ovoid hypoattenuating mass located in the mid esophageal wall (arrow). The presence of calcifications within this mass is highly suggestive of a leiomyoma

On CT, it appears as an ovoid homogeneous mass with a smooth surface within the esophageal wall. Calcifications may be present, which are highly suggestive of the diagnosis [35] (Fig. 14). Diffuse moderate enhancement occurring post contrast and evidence of local invasion should raise the possibility of leiomyosarcoma.

### Esophageal cancer

#### Squamous cell carcinoma

**Fig. 15:**
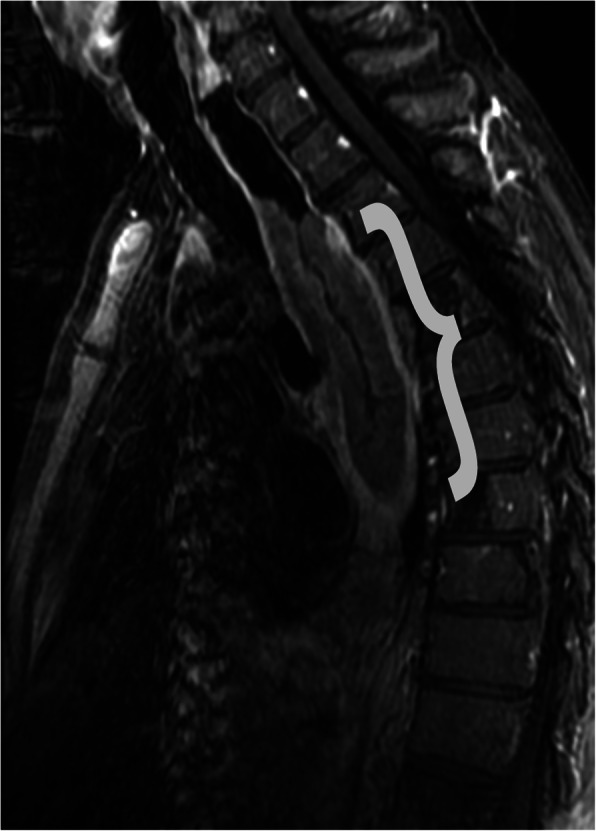
Squamous cell carcinoma. Sagittal T1WI MR post contrast image showing an area of irregular thickening of the upper to mid esophagus with peripheral irregular enhancement (bracket) in the setting of biopsy proven squamous cell carcinoma of the esophagus

Like SCC of the lung, SCC of the esophagus is a malignant tumor characterized by the presence of keratinization and/or intercellular bridging by tumor cells and epithelial cells. It is the most common form of malignant esophageal tumor worldwide, however, has been decreasing in incidence in the USA. The major risk factors include tobacco and alcohol, nutritional deficiencies, and geographic location [37]. Patients typically present with progressive dysphagia, painful swallowing, weight loss, and chest pain, symptoms which typically indicate advance or invasive disease. Barium esophagram will demonstrate an infiltrative lesion with irregular stricture of the lumen and abrupt shouldering, with possible areas of ulceration. Superficial lesions can be difficult to visualize, with a plaque-like, polypoid, or ulcerative appearance [38]. CT imaging typically will show an irregular mass arising from the esophageal wall versus eccentric/localized thickening of the esophageal wall ≥ 5 mm, with circumferential wall thickening occasionally occurring (Fig. 15). Fat stranding of the adjacent soft tissues is suspicious for invasion. It is important to note that these lesions can be easily overlooked if the lesion is small and/or the esophageal lumen is not well distended. CT plays a critical role in identifying advanced disease and predicting possible treatment options for example respectability, as well as identifying complications associated with the tumor and its treatment, including tracheoesophageal fistula formation and esophageal obstruction [37]. On MR imaging, the esophageal lesion shows T2WI hyperintensity and is similar to CT in determining invasion, lymph node involvement, and distant metastasis [38]. SCC of the esophagus and its metastasis will show avid uptake on FDG-PET/CT [38].

## Musculoskeletal

Neoplasms of the musculoskeletal system can arise from either the bones or the soft tissues, including the muscles and cartilage. Neoplasms of the thoracic inlet can arise from the lower cervical/upper thoracic spine, ribs, neck muscles, cartilage, and subcutaneous fat. Malignant lesions of the musculoskeletal system typically get a prefix based on the tissue type the tumor is composed of and the suffix of sarcoma. Some of the more commonly encountered thoracic inlet neoplasms of the musculoskeletal system include osteochondroma, lipoma, liposarcoma, and Ewing sarcoma.

### Osteochondroma

**Fig. 16:**
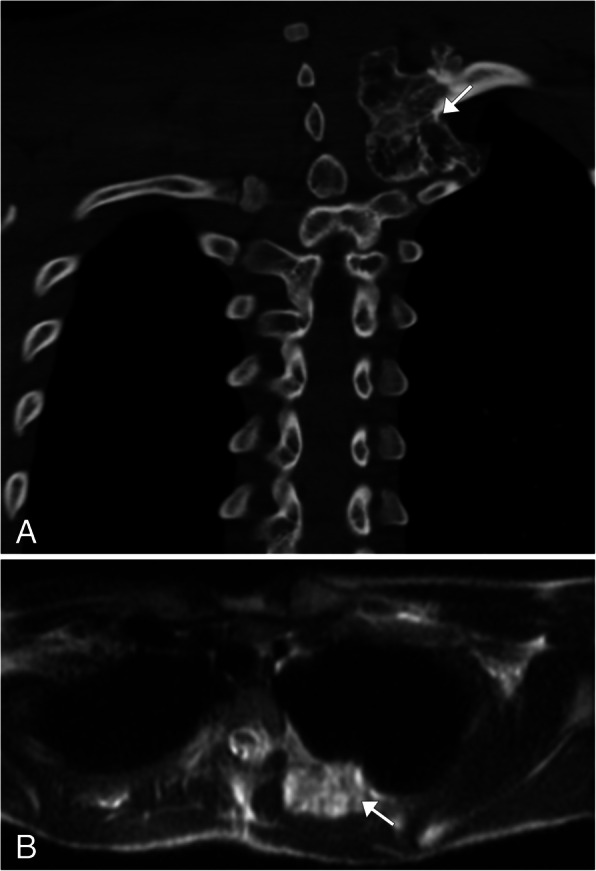
Osteochondroma. **a** Coronal CT image of the chest showing an exostosis involving the medial aspect of the posterior first rib (arrow). **b** Axial T2WI MR imaging demonstrating a hyperintense continuous hyaline cap (arrow). There was no evidence of adjacent soft tissue involvement

Osteochondroma is a commonly encountered benign tumor of the bone, accounting for 15% of all bone tumors, primarily composed of cortical and medullary bone with an overlying hyaline cap. Any bone that matures from an endochondral ossification can develop an osteochondroma, as these lesions typically arise from a separated portion of the growth plate that continues to grow independently away from the adjacent joint. While these lesions are often asymptomatic and incidentally detected, symptoms when present typically occur secondary to the location of the lesion and the resulting mass effect on adjacent structures, fracture/injury of the lesion, and very rarely secondary to malignant degeneration. While the majority of these lesions occur in the extremities away from the thoracic inlet, these tumors can arise from the posterior spinal elements, scapula, and rib surfaces, especially favoring the costochondral junctions [29]. CT and plain film imaging will demonstrate a lesion that has a medullary cavity that is contiguous with the parent bone, a finding which is easiest to see in lesions arising from the long bones, however, can be difficult to identify in lesions arising from the flat bones (e.g., scapula and spine) (Fig. 16a). MR is the most ideal modality to identify and evaluate the hyaline cap [29]. MR imaging demonstrates both the cortical and medullary continuity, and the non-mineralized hyaline cap will have an intermediate to low signal intensity on T1WI and high signal intensity on T2WI (Fig. 16b). In addition, MR can evaluate for injury/edema of the adjacent bone and soft tissues, as well as mass effect on the adjacent tissues [39]. Malignant degeneration should be suspected in lesions that have a hyaline cartilage cap greater than 1.5 cm in thickness or a growing cap. Nuclear medicine bone scintigraphy directly correlates with the degree of endochondral bone formation, with increased uptake in younger patients. Ultrasound can also be used to obtain accurate measurements of the hyaline cartilage cap thickness if sonographically accessible.

### Lipoma

**Fig. 17:**
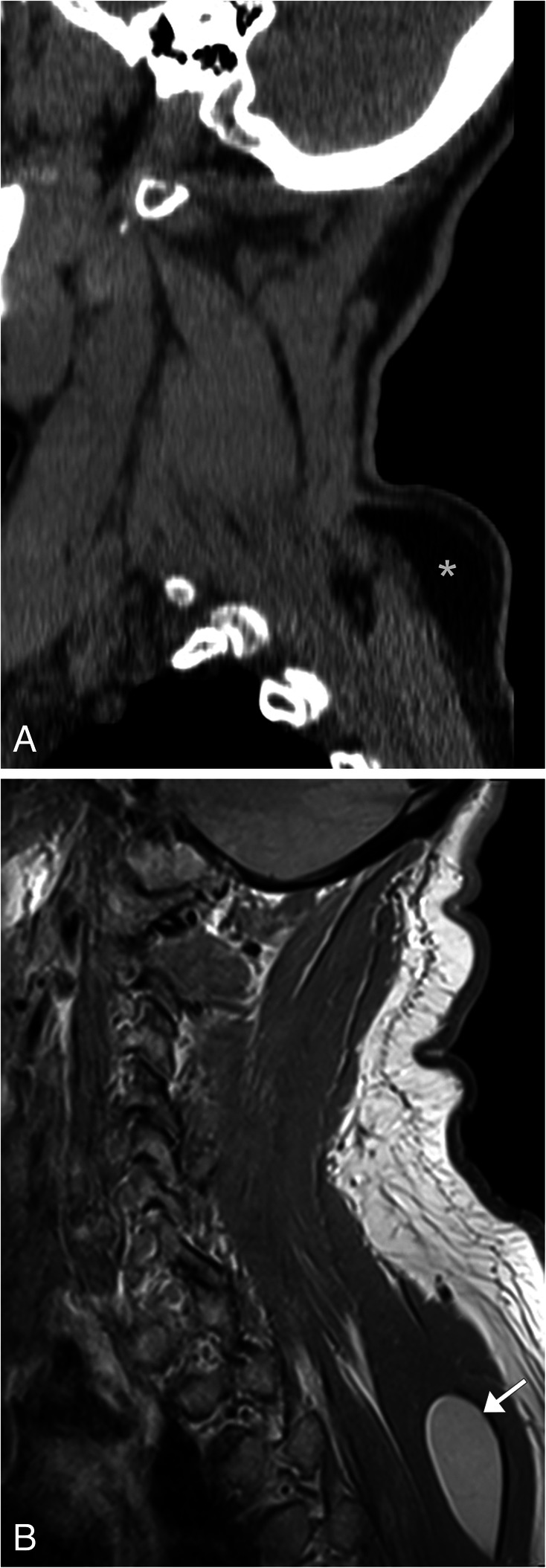
Lipoma. **a** Sagittal CT image of a companion case demonstrating a low-density lesion (*) measuring between − 65 and − 120 Hounsfield units in the posterior superficial soft tissue of the neck at the level of C7–T1. **b** Sagittal T1WI MR of the cervical spine shows a hyperintense lesion located between the posterior spinal muscles at the level of the upper thoracic spine (arrow), similar in signal to the subcutaneous fat

Lipoma is one of the most common benign soft tissue masses encountered clinically. It is composed of homogeneous mature adipose tissue that is typically surrounded by a fibrous capsule. These lesions are often subcutaneous in location, categorized as superficial or deep depending on its position relative to the muscle fascia. Lipomas can also occur within the muscle, bone, pleura, or other structures including the esophagus. While often asymptomatic, the lesions may present as a painless mass, and large lesions can become painful if there is compression of an adjacent nerve [40]. On ultrasound, lipomas have variable echogenicity, typically hyperechoic to isoechoic, with similar appearance to the adjacent subcutaneous fat, and the echogenic capsule is often difficult to identify. Lipomas on CT and MR have a pathognomonic appearance, mirroring the subcutaneous fat. On CT, lipomas are homogenous fat density masses measuring between − 65 and – 120 HU (Fig. 17a). On MRI, these masses will be T1WI and T2WI hyperintense, suppress on fat–saturated images, and have minimal to no enhancement post contrast [41] (Fig. 17b).

### Liposarcoma

**Fig. 18:**
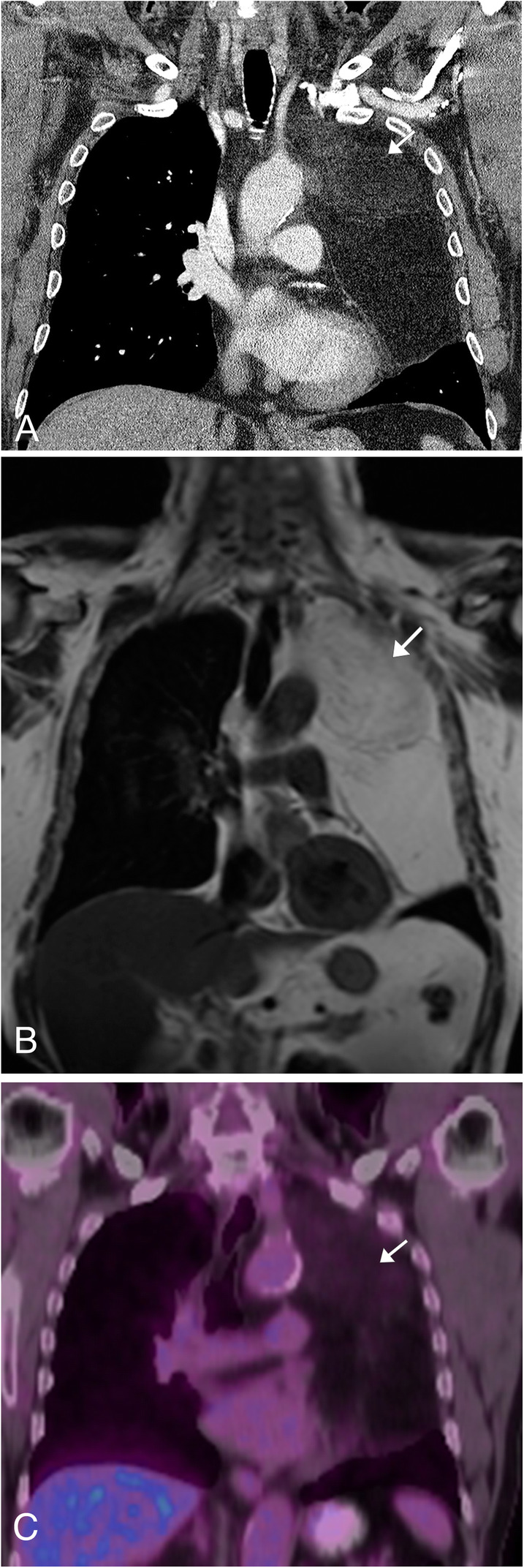
Liposarcoma. **a** Coronal CT image post contrast demonstrates a large low attenuation mass (fatty) located in the left upper hemithorax. The mass contains a heterogeneous area increased density/soft tissue component in the superior aspect of the mass (arrow), a finding which is suspicious for a liposarcoma. **b** Coronal T1WI MR images of the same lesion demonstrating a hyperintense mass in the left hemithorax with areas of heterogeneous low signal in the upper aspect of the mass (arrow). **c** Coronal FDG-PET/CT of the same lesion showing mild increased hypermetabolism in the area of heterogeneous enhancement within the fatty mass (arrow)

Liposarcoma is the malignant equivalent to the benign lipoma, also arising from fatty mesenchymal cells. It is the second most common form of soft tissue sarcoma in adults, accounting for 20–25% of adult soft tissue sarcomas [42]. Symptoms at presentation are often dependent on the size and location of the lesion, extending from asymptomatic, to a painless palpable lump, to vague discomfort. Prognosis is dependent on the subtype of the tumor, ranging from indolent non-metastasizing disease to aggressive lesions with metastasis. These masses can occur anywhere in the body, but are most frequent in the extremities and retroperitoneum. Whereas lipomas are homogenously fatty masses, liposarcoma should be suspected if heterogeneity, enhancement, or prominent soft tissue components are present within a fatty mass. The appearance of liposarcomas also depends on the subtype [29]. Well-differentiated liposarcomas are typically composed of primarily fatty tissue (about 50 to 75%) with bands of thickened septations and/or nodular soft-tissue, which typically enhance post contrast (Fig. 18). An increasing ratio of the soft tissue to fatty component often indicates a more aggressive/higher grade of liposarcoma [29]. Liposarcoma often has an unpredictable growth pattern and infiltrative appearance, making it difficult to assess on follow up imaging.

### Ewing sarcoma

**Fig. 19:**
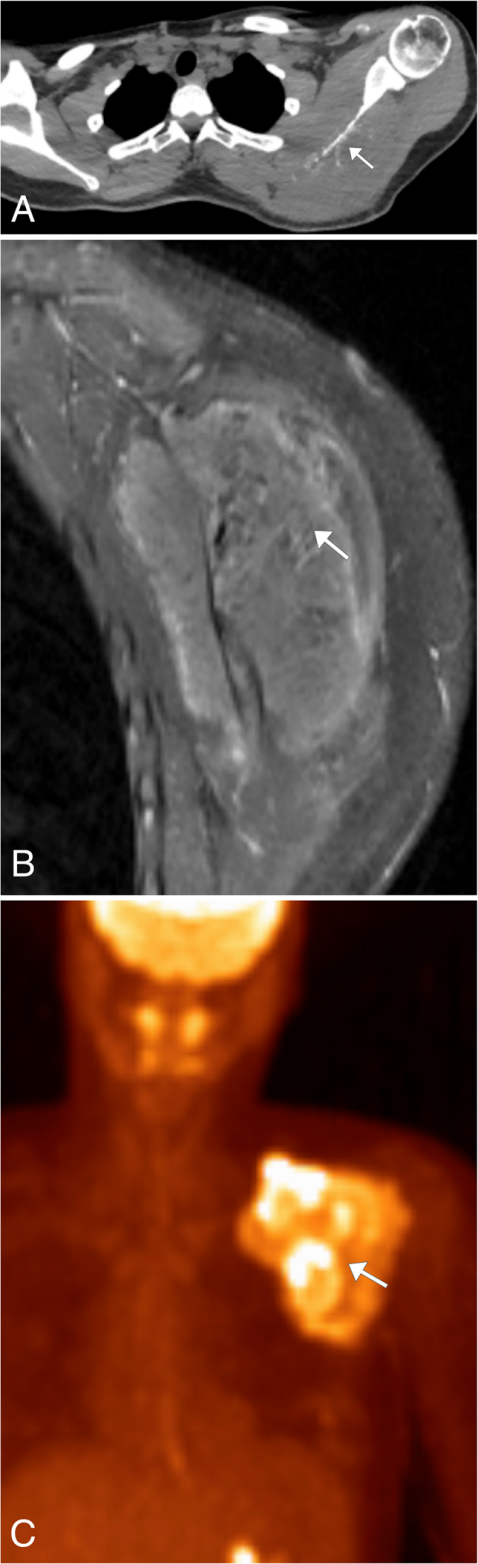
Ewing sarcoma. **a** Axial noncontrast chest CT demonstrates permeative destructive changes of the left scapula (arrow) with the large soft tissue component of the mass not well seen. **b** Sagittal T1WI MR image post contrast allows for visualization of the associated enhancing mass (arrow) which involves the surrounding left scapular soft tissues and musculature. **c** Coronal FDG-PET/CT image demonstrating the hypermetabolic nature of the lesion associated with the left scapula (arrow)

Ewing Sarcoma is the second most common primary malignant tumor of bone in children and adolescents [43]. While the tumor most commonly affects lower limbs and pelvis, involvement of the upper limbs, spine, and ribs can occur at the level of the thoracic inlet. Patients typically present with nonspecific discomfort, occasionally associated with a palpable mass or swelling. Additional symptoms will be related to the location of the tumor, such as pleuritic pain in chest wall masses and neurologic symptoms in spinal masses. On radiography and CT imaging, Ewing sarcoma shows clearly aggressive features, including lytic bone destruction with a moth-eaten appearance and a wide zone of transition, as well as osseous sclerosis [44] (Fig. 19a). CT imaging will also show the associated soft tissue mass which is typically homogeneous with the same attenuation as the adjacent muscle. Post contrast CT images demonstrates diffuse or peripheral nodular enhancement [44]. MR imaging is the modality of choice to best image the full extent of the tumor. The tumor will have homogeneous intermediate signal on T1WI, heterogeneous intermediate to high signal on T2WI, and heterogeneously enhance post contrast (Fig. 19b). MR can determine the amount of marrow replacement and cortical destruction in the osseous portion of the lesion [44]. FDG-PET/CT is utilized for staging, restaging, and treatment planning, as these tumors show increased metabolic activity with a mean SUV of 5.3 [43] (Fig. 19c).

## Endocrine

The thyroid and parathyroid glands are located within the thoracic inlet and are the most common sources of both benign and malignant neoplasms of the endocrine system. Some of the more commonly encountered thoracic inlet neoplasms of the endocrine system include parathyroid adenoma, thyroid adenoma, paraganglioma, and thyroid carcinoma.

### Parathyroid adenoma

**Fig. 20:**
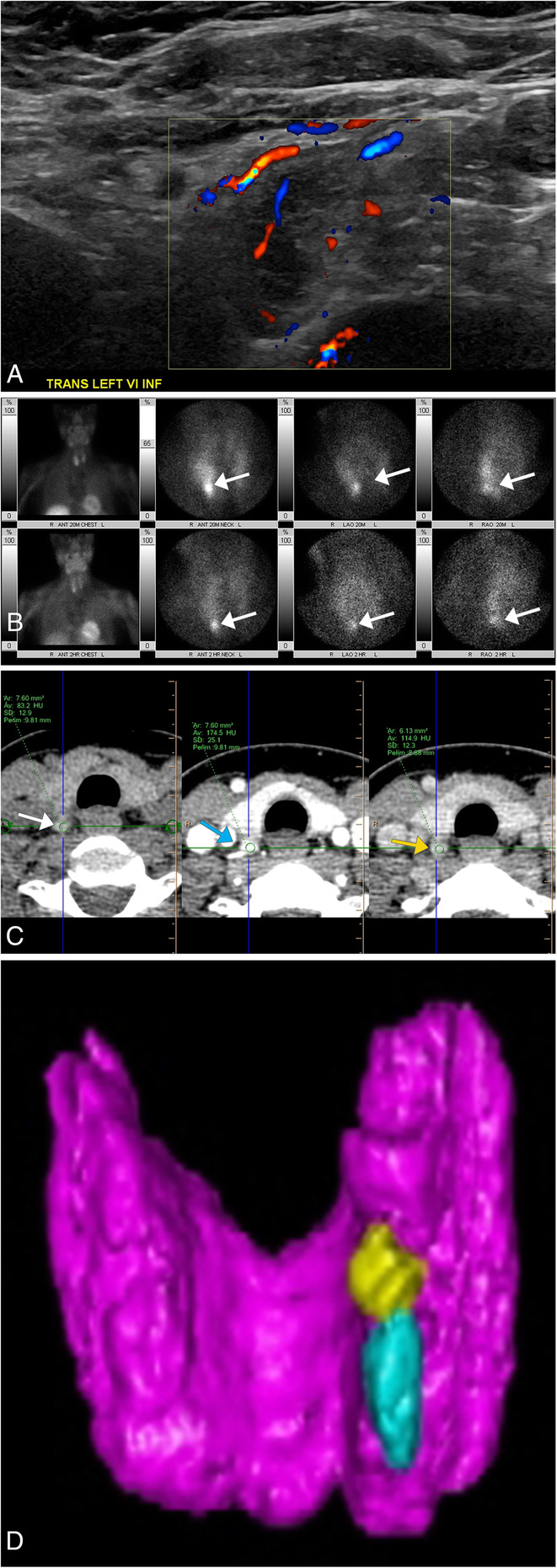
Parathyroid Adenoma. **a** Grayscale with Doppler ultrasound image demonstrates an ovoid well-circumscribed hypoechoic lesion containing cystic anechoic areas, located at the inferior posterior aspect of the left thyroid lobe. Doppler shows the vessels feeding the adenoma. **b** Tc99 sestamibi scan demonstrates increased radiotracer uptake in the lower pole of the right thyroid lobe in a companion case with persistent uptake 2 h later (arrows). Multiphasic 4-dimensional CT imaging of the parathyroid glands was performed in the same patient as the nuclear medicine study. **c** Axial precontrast (left), arterial phase (middle), and late arterial/delayed phase (right) images identified two enhancing nodules in the posterior aspect of the right lower thyroid lobe which washed out on delayed phase imaging (upper nodule shown). This nodule measured 83 HU precontrast (white arrow), 175 HU on arterial phase (blue arrow), and 115 HU on delayed phase (orange arrow). **d** 3D reconstructions created from the CT imaging demonstrate the position of the two nodules (yellow and turquoise) relative to the thyroid gland (purple) on the posterior view, assisting in surgical pre-procedure planning

Parathyroid adenoma is a benign tumor of the parathyroid gland and is the most frequent source of primary hyperparathyroidism. The tumor secretes an abundance of parathyroid hormone (PTH) resulting in elevated calcium levels. Patients complain of osteoporosis (painful bones), renal calculi (stones), peptic ulcers (abdominal groans), fatigue, depression, and mental status changes (moans). On ultrasound, parathyroid adenomas will appear as a small well-circumscribed, ovoid, solid, homogenous, and hypoechoic lesion, typically located posterior and/or inferior to the adjacent thyroid lobe [45] (Fig. 20a). Doppler may show a feeding vessel that first branches peripherally around the gland before entering the adenoma at a pole, creating a characteristic rim of vascularity. Dual–phase technetium (Tc)-99 m sestamibi parathyroid scintigraphy can be used to identify adenomas that are not localized by ultrasound, especially those that may be ectopically located, with early images showing uptake in the thyroid gland, and the delayed images demonstrating washout from the thyroid and persistent uptake in the adenoma [46] (Fig. 20b). Multiphasic 4-dimensional CT imaging has superior detection of parathyroid adenomas when compared to ultrasound and sestamibi imaging, including identifying ectopic lesions, and provides a detailed anatomic look at the location of the parathyroid adenoma in relationship to adjacent structures in the neck and mediastinum (Fig. 20c, d). This modality takes advantage of the brisk wash-in and washout enhancement pattern of the parathyroid gland as compared to the surrounding structures, including the adjacent thyroid gland and lymph nodes [45]. On MRI, parathyroid adenomas are isointense to hypointense relative to the surrounding muscle on T1WI and hyperintense on T2WI [45].

### Thyroid adenoma

**Fig. 21:**
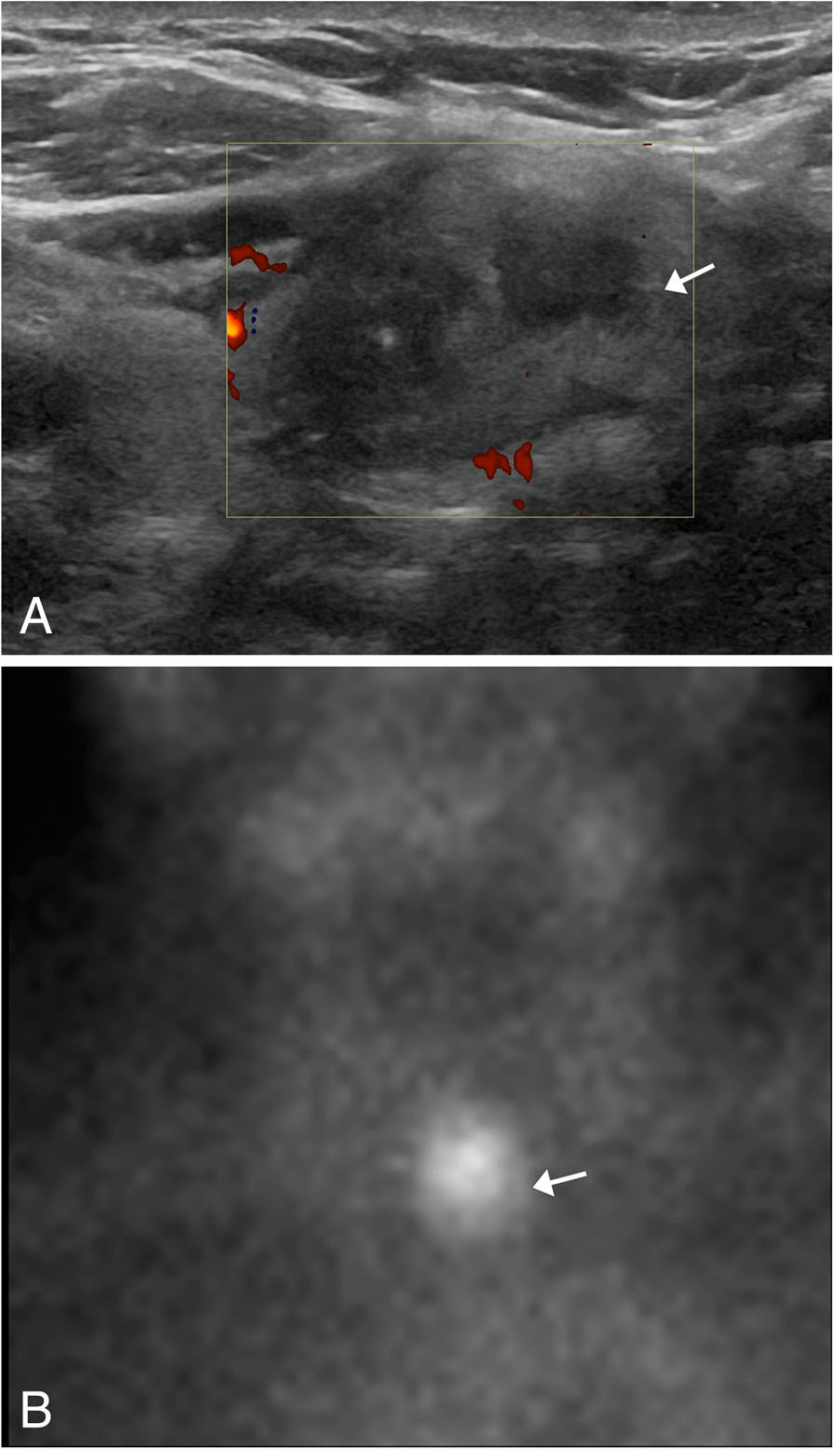
Thyroid adenoma. **a** Grayscale ultrasound image shows an oval nodule with heterogeneous internal echoes and cystic components without internal flow (arrow). **b** Iodine 123 thyroid scan shows persistent increased radiotracer uptake within the nodule on the 24-h delayed images (arrow), findings consistent with an hyperactive thyroid adenoma (hot nodule)

Thyroid adenoma is a benign neoplasm of the thyroid gland that is composed of differentiated follicular cells with a surrounding fibrous capsule. Patients are typically asymptomatic; however, a painless palpable nodule is occasionally identified on physical exam. Imaging features will be dependent on the make-up of the gland, as calcification, ossification, necrosis, cystic degeneration, fibrosis, and hemorrhage are often present to a variable degree. On ultrasound, findings that suggest a benign nodule include a predominantly isoechoic, cystic, or mixed (spongiform) appearance (Fig. 21a). On Doppler, adenomas can be avascular or have a primarily peripheral blood vessel that can be seen extending towards the center of the adenoma in what is known as the spoke wheel pattern [47]. Thyroid scintigraphy can be helpful in classifying the nodule based on activity; if hyperfunctioning, it is known as a hot nodule, and if it is non-functioning, it is known as a cold nodule (Fig. 21b). Classifying the nodules helps set the course for additional work up. Thyroid adenomas are often hypo- to isoattenuating and may be calcified on CT imaging. On MRI, these nodules are commonly iso- to hypointense on T1WI and hyperintense on T2WI and can enhance post contrast administration [48]. Because most of these lesions are found incidentally, the ACR has published a consensus for management of incidental thyroid nodules, which gives guidelines about which nodules need further follow up and which do not. They established the 3 tiers that depict the need of further evaluation: (1) high-risk imaging characteristics including a suspicious lymph nodes measuring 1.5 cm short axis, local invasion, and/or PET avid nodule; (2) nodule measuring > 1 cm in a patient younger than 35 years; (3) and nodules > 1.5 cm in a patient older than 35 years of age. Patients that meet any of these criteria should have a dedicated thyroid ultrasound for further evaluation [49]. Another factor that should also be taken into consideration is the patient’s life expectancy, which is based on the patients’ age and comorbidities. If the patient has a low life expectancy and does meet one of the 3 tiers, follow-up imaging is not recommended [49]. The 3 tiers have also been slightly modified depending in what modality the incidental thyroid nodule is found. For CT and MRI, the previously mentioned 3 tiers are applied. For nuclear medicine studies, such as PET, increased uptake of a nodule is very suspicious of malignancy. For this reason, it is recommended that a PET avid lesion in a person with general population risk should have an ultrasound evaluation and an FNA [49, 50]. For lesions incidentally detected on neck ultrasound (not dedicated thyroid imaging), the 3-tier criteria apply. Suspicious features that must be evaluated in these nodules are microcalcification, marked hypoechogenicity, irregular margins, and taller than wide shape in transverse view [49].

### Paraganglioma

**Fig. 22:**
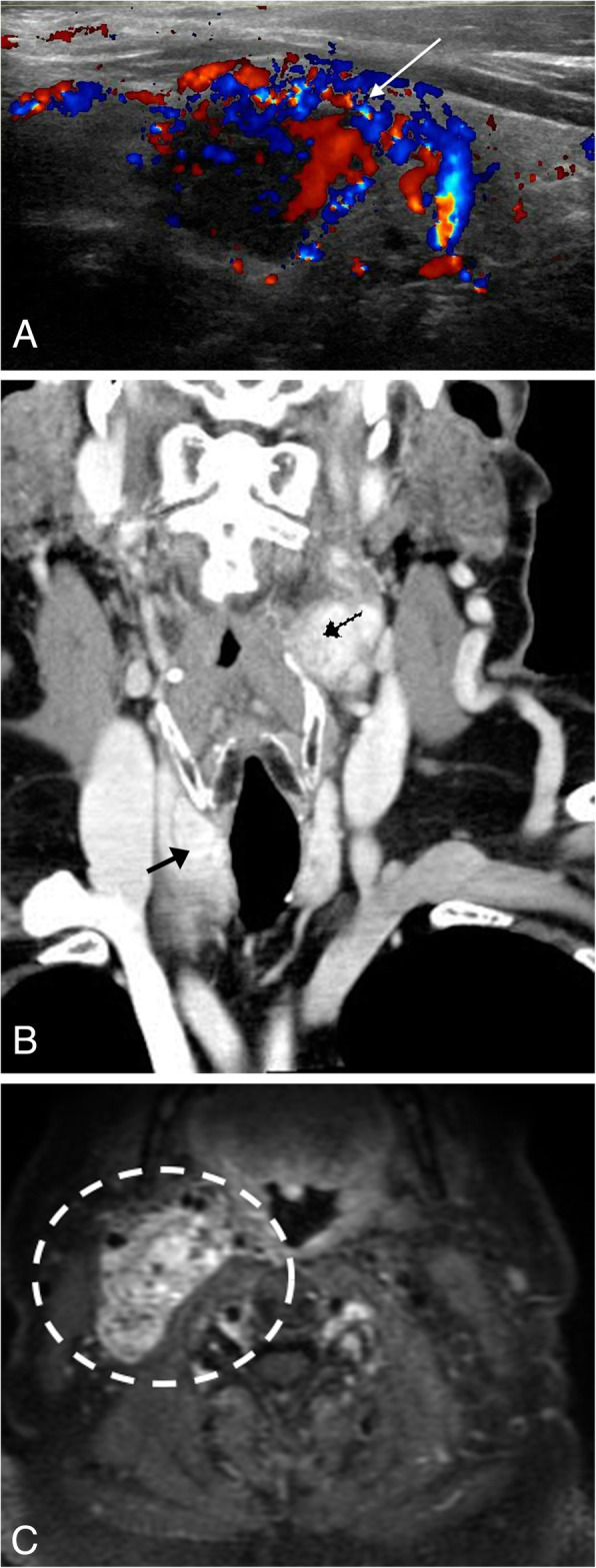
Paraganglioma. **a** Ultrasound Doppler image shows a hypervascular (arrow) lesion within the right lobe of the thyroid. **b** Coronal CT image post contrast demonstrates an enhancing lesion within the medial aspect of the right lobe of the thyroid (solid arrow) and in the left carotid space (dashed arrow), both of which were biopsied and shown to be paragangliomas. **c** Axial T2WI MR of the neck base in a companion case demonstrates the classic salt and pepper appearance of the lesion. The high T2 signal (salt) corresponds to the areas of hemorrhage, and the low signal (pepper) corresponds to the flow voids within the lesion

Paraganglioma is a rare neuroendocrine tumor arising from the paraganglia, including the paraganglia of the head and neck and the mediastinum. Most of these tumors arise in the fourth and fifth decades of life and clinical presentation is typically dependent on the functionality of the tumor [51]. Functional paragangliomas secrete an excess of catecholamine, with the patient presenting with headache, palpitations, and sweating. Nonfunctioning paragangliomas often present as a slow growing palpable mass or pain related to local growth. Head and neck paragangliomas are typically parasympathetic in origin and non-secretory, including those that arise from the vagus nerve and glossopharyngeal nerve. Thoracic/mediastinal paragangliomas can be parasympathetic in origin (if they arise in the anterior mediastinum) or sympathetic (if it arises from the posterior mediastinum) [51]. This tumor appears as a well-defined soft tissue mass located along the distribution of a nerve (Fig. 22a, b). On CT imaging, the lesion will avidly enhance post contrast and demonstrate delayed washout due to the increased vascularity. MRI is the reference exam for the evaluation of paragangliomas. On T1W1, the lesion is solid and isointense, and T2WI demonstrates the classic salt and pepper appearance: the “pepper” appearance is due to the low signal flow voids from the high flow vessels within the lesion, and the “salt” corresponding to the high intensity signal due to areas of hemorrhage [51] (Fig. 22c). On post contrast images, there is intense enhancement, which may be homogenous or heterogeneous depending on the presence of necrotic tissue [52]. Nuclear medicine imaging can be used to target the tumor-specific catecholamine production, such as an Octreoscan [52].

### Thyroid carcinoma

**Fig. 23:**
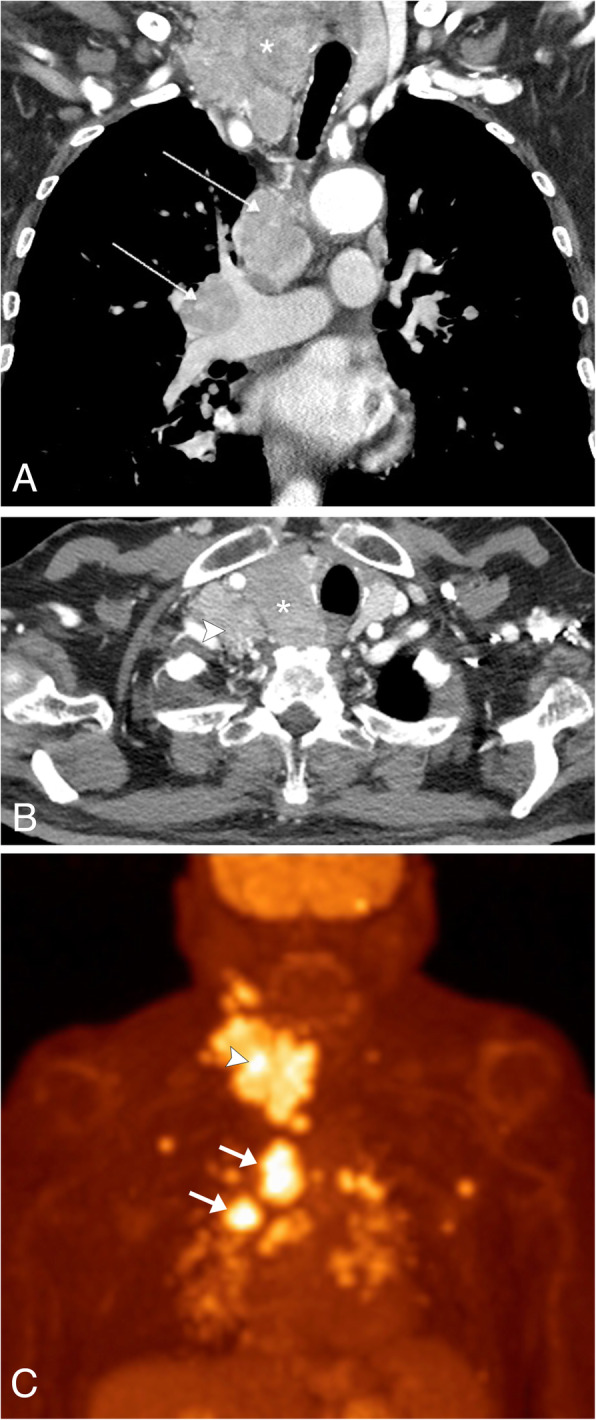
Thyroid CA. Contrast-enhanced CT coronal (**a**) and axial (**b**) images demonstrate a large mass in the right lower neck associated with an enlarged and heterogeneous right thyroid lobe (asterisk), which is causing leftward shift of the trachea. Enlarged right neck base, mediastinal and right hilar lymph nodes (arrows) are noted. Biopsy of these lesions confirmed the presence of metastatic thyroid cancer. **c** Coronal FDG-PET/CT shows avid hypermetabolic uptake in the right neck base mass (arrowhead) and within the metastatic lymph nodes within the neck and chest (arrows)

There are four major subtypes of primary thyroid carcinoma: papillary, follicular, medullary, and anaplastic. The most common subtype is papillary thyroid cancer, accounting for 70–75% of primary thyroid cancers [53]. Patients typically present with a palpable mass in the neck base at the level of the thyroid gland, and local lymph node involvement is commonly present on initial evaluation [54]. Nodal metastatic disease is often cavitary with cystic components, thickened walls, septations, mural nodules, and punctate calcifications. Ultrasound will show an irregular mass of the thyroid gland, typically located in the subcapsular region, typically with internal vascularity. Small echogenic foci are commonly present, correlating with microcalcifications [53]. CT and MR imaging are useful to demonstrate the extrathyroidal extension of the tumor and can determine the full extent of large thyroid cancers, including identification of abnormal lymph nodes for staging purposes (Fig. 23a, b). On MR imaging, the cystic components will demonstrate fluid signal, and the solid components will be hypointense to muscle on T1WI and variable signal on T2WI [55]. The lesions are often FDG-PET/CT avid and are a common incidental finding on PET imaging (Fig. 23c). Thyroid cancer has a higher SUV compared to benign thyroid lesions; however, there is an overlap and for this reason, further evaluation of these lesions is needed typically with ultrasound and biopsy [55]. Nuclear medicine ^131^I/^123^I whole body scan can be used to detect residual cancer in the resection site and distant metastasis [55].

## Vascular

Neoplasms of the vascular system are a complex group of lesions that can arise from the vessels, including the arteries, veins, and capillaries. These neoplasms are often confused (both medically and historically) with vascular malformations as they can have similar imaging appearances and clinical presentations.

### Intraosseous venous vascular malformations

**Fig. 24:**
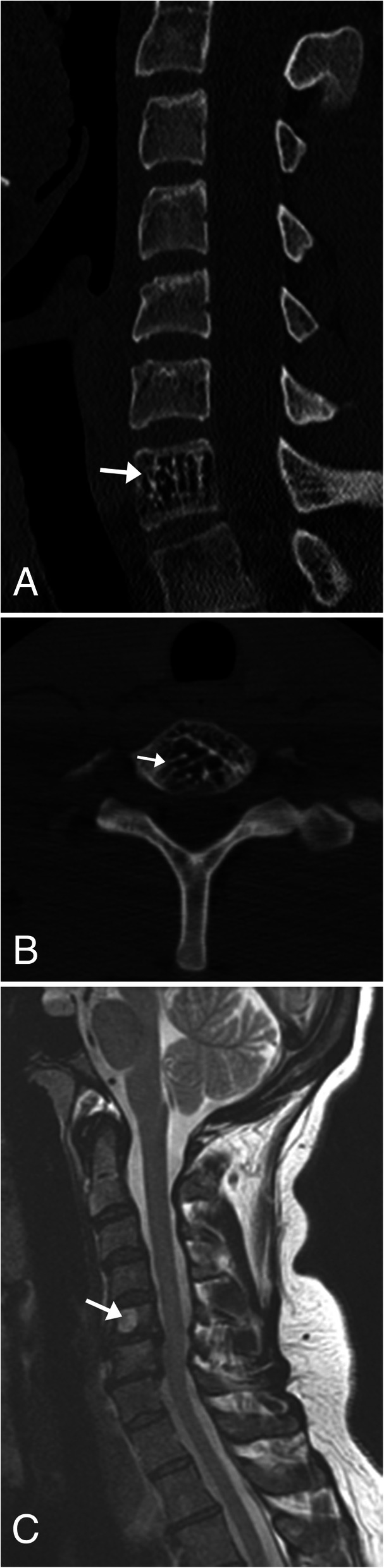
Intraosseous venous vascular malformation (formerly hemangioma). **a** Sagittal and (**b**) axial CT of the cervical spine showing the thickened vertical trabeculation (arrow) in a background of fatty tissue classically seen in the lesion at C7, described as the “corduroy sign” in the sagittal or coronal planes and a polka-dot appearance in the axial plane. **c** Sagittal T2WI MR image of the cervical spine in a companion case demonstrates the hyperintense nature of a lesion in the C5 vertebral body due to a fat component

Intraosseous venous vascular malformations, formerly called hemangiomas, are commonly and incidentally encountered lesions of the vertebral bodies [56]. The lesion is discussed here due to its former name implicating it as a neoplasm. Histologically, the lesion contains multiple small vessels scattered among the trabecula of bone and may also contain elements of fat, smooth muscle, and fibrous tissue which can make the lesion expansile [57]. While most commonly asymptomatic, symptoms if present are often related to mass effect (due to extension into the spinal canal or neural foramen), with patient’s presenting with pain, myelopathy, and radiculopathy [57]. Interestingly, pregnancy can contribute to the development of aggressive or symptomatic lesions, likely due to hormone stimulation. On CT and radiographs, intraosseous venous vascular malformations have thickened vertical trabeculations in a background of fatty tissue, described as the corduroy sign on coronal and sagittal imaging and a polka-dot appearance on axial imaging [56, 57]  (Fig 24a, b). On MRI, these lesions are typically hyperintense on T1WI due to fat content and have hyperintense signal on T2WI; fat-saturated images can help to confirm the presence of fat. The lesions will enhance with contrast on CT and MRI [57] (Fig. 24c).

## Conclusion

The thoracic inlet is a critical landmark in radiology, as it is a central conduit for many organ systems, and therefore can contain a vast array of neoplastic pathology. It is therefore important to thoroughly evaluate this region and be aware that it may be overlooked on imaging of the neck or chest. When evaluating for neoplastic lesions of the thoracic inlet on both chest and neck imaging, it is important to tackle this region with a methodical plan in order to reduce the chance of missing clinically important findings. We therefore propose a system-based approach to the imaging of the thoracic inlet based on the body systems present in this location. The systems to consider in this region are respiratory, lymphatic, neurologic, enteric, musculoskeletal, endocrine, and vascular. A thorough knowledge of the regional/system-based anatomy can therefore help the radiologist correctly identify neoplastic lesions. 

## Data Availability

Not applicable

## Abbreviations

AIS: Adenocarcinoma in situ
CT: Computed tomography
FDG: 2-[Fluorine-18] fluoro-2-deoxy-d-glucose
HPV: Human papilloma virus
HU: Hounsfield units
MIA: Minimally invasive adenocarcinoma
MPNST: Malignant peripheral nerve sheath tumor
MR: Magnetic resonance
NF1: Neurofibromatosis type 1
NF2: Neurofibromatosis type 2
PTH: Parathyroid hormone
SCC: Squamous cell carcinoma
US: Ultrasound
